# TRPA1 Channel Activation Inhibits Motor Activity in the Mouse Colon

**DOI:** 10.3389/fnins.2020.00471

**Published:** 2020-05-27

**Authors:** Abdul-Azim Hassan, Ben Sleet, Zoe Cousins, Chris David Keating

**Affiliations:** Department of Pharmacy, Pharmacology and Postgraduate Medicine, University of Hertfordshire, Hatfield, United Kingdom

**Keywords:** TRPA1, motility, colon, HNE (4-hydroxy-2-nonenal), enteric nervous system, transient receptor channels

## Abstract

There is a growing awareness of the role that TRP channels play in regulating sensory and motor functions in the gastrointestinal tract. In this study we used an *in-vitro* murine model of colonic peristaltic-like complexes (CPMCs) to evaluate the role of exogenous and endogenous TRPA1 signaling processes in regulating colonic motility. Using *in-vitro* recordings of intraluminal pressure to monitor the presence of CPMCs in colonic segments we performed a series of experiments on male CD1 mice (2 months of age) and found that CPMC activity was attenuated by TRPA1 agonists. Bath application of the TRPA1 antagonist HC-030031 had no effect upon basal CPMC activity whereas application of the synthetic TRPA1 agonist ASP7663 caused a reversible dose dependent decrease in CPMC frequency that was blocked by HC-030031. Cinnamaldehyde and 4-hydroxy-2-nonenal elicited long lasting decreases in CPMC frequency that were blocked by HC-030031 whereas the decreased CPMC activity invoked by AITC could not be blocked by HC-030031. Our results show that any potential mechanosensory function of TRPA1 doesn’t involve contributing to distension induced colonic motor activity and that a role for TRPA1 in the colon is through regulating motility through exogenous and endogenous agonist induced inhibitory effects.

## Introduction

The transient receptor potential (TRP) family is a group of excitatory cation channels expressed in multiple cell types within the body that act as environmental sensors to noxious chemical, thermal and mechanical stimuli (Ramsey et al., 2006). An important member of this super family is the transient receptor potential ankyrin 1 (TRPA1), initially described as a cold sensitive non-selective cation channel, but now acknowledged to have multiple physiological roles (Story et al., 2003; Bandell et al., 2004; Nagata et al., 2005). In addition to thermal stimuli, TRPA1 has been shown to be activated by a wide range of chemicals including nutrient derived compounds such as allyl isothiocyanate (AITC), cinnamaldehyde (CMA), and allicin that are found in mustard, cinnamon and garlic, respectively, as well as acrid chemicals including acrolein and tear gas. These compounds are believed to activate the channel through a process of modifying specific cysteine or lysine residues contained within the amino terminus of the protein (Hinman et al., 2006; Macpherson et al., 2007; Clapham, 2015). In addition to exogenous agonists, TRPA1 can also be activated by endogenous chemicals including 4-hydroxy-2-nonenal (HNE), an inflammatory mediator produced through oxidative stress (Esterbauer et al., 1991) providing evidence that TRPA1 is capable of transducing inflammatory derived signals into cellular responses (Trevisani et al., 2007).

Within the gastrointestinal tract TRPA1 is expressed on the terminals of visceral sensory afferents and acts as a mechanotransducer. TRPA1 activation is thought to be a contributing factor in the generation of afferent mechano- hypersensitivity seen in models of colitis (Brierley et al., 2009). This role of TRPA1 is enhanced in inflammatory conditions associated with visceral hyperalgesia suggesting that a function of TRPA1 is as a sensor of inflammation. TRPA1 is also activated by the release of inflammatory mediators when tissue injury occurs or in diseased states. This dual function of TRPA1 as a detector and instigator of inflammatory agents makes TRPA1 a “gatekeeper” of chronic inflammatory disorders (Bautista et al., 2013).

TRPA1 is also expressed in enterochromaffin (EC) cells in the enteric mucosa where they exhibit a graded expression profile along the length of the gut and are absent from the colon (Cho et al., 2014). TRPA1 is also expressed within myenteric neurons of the gastrointestinal tract where their expression is strongest in nitrergic neurons the colon (Poole et al., 2011). The broad expression profile and promiscuous activation of this channel suggests that TRPA1 is uniquely positioned to respond to luminal or inflammatory stimuli and is involved in mediating a wide range of physiological and pathophysiological events in the gastrointestinal tract. TRPA1 activation gives rise to varied physiological responses depending on the region of gut exposed to TRPA1 compounds, and their route of administration. Application of TRPA1 agonists to myenteric neurons activates them: luminal application of TRPA1 agonists in the small intestine is excitatory, whereas serosal application in the colon attenuates spontaneous contractile behavior (Poole et al., 2011; Tsuchiya et al., 2016). Activation of TRPA1 channels led to changes in contractile behavior of isolated intestinal segments (Penuelas et al., 2007) and *in-vivo* changes in gastric emptying and colonic motility (Doihara et al., 2009a, b). Activation of TRPA1 channels located on enterochromaffin (EC) cells have been shown to have important roles in EC cell signal transduction (Nozawa et al., 2009; Bellono et al., 2017). Together these findings support a role for TRPA1 in mediating motor responses of the large intestine.

However, there is little information as to the specific effects of TRPA1 activation on peristaltic activity in the gut. In this study we aimed to address this question using an *in-vitro* model of peristaltic-like behavior (Keating et al., 2014). This model employs segments of mouse colon which generate robust and stereotypic patterns of colonic peristaltic motor complexes (CPMCs) when mounted in an organ bath and perfused with Krebs solution (Abdu et al., 2002). The advantage of this system is that the intestinal segments contain all the functional components of the enteric nervous system necessary to initiate peristaltic-like motor activity (Brookes et al., 1999) and that CPMC activity is similar to the intestinal motor patterns observed *in vivo*. For this study we assessed CPMC characteristics (frequency and amplitude) using compounds with known effects upon TRPA1 channel pharmacology as a way of addressing the role of this channel in regulating motility like behavior in the murine colon.

## Materials and Methods

### Animals

All experiments were performed at the University of Hertfordshire. Fifty one adult male mice (CD1, Charles River, United Kingdom), 6–8 weeks old and weighing ~30 g were used. Animals were housed under controlled ambient temperature (21 ± 2°C) and light-dark cycle (12:12 h) and were allowed free access to food and water. Animals were euthanized by a gently rising concentration of carbon dioxide followed by cervical dislocation. Animals were maintained in accordance with Home Office regulations following the Animal and Scientific Procedures Act 1986.

### Motility Recordings in the Mouse Colon

**FIGURE 1:**
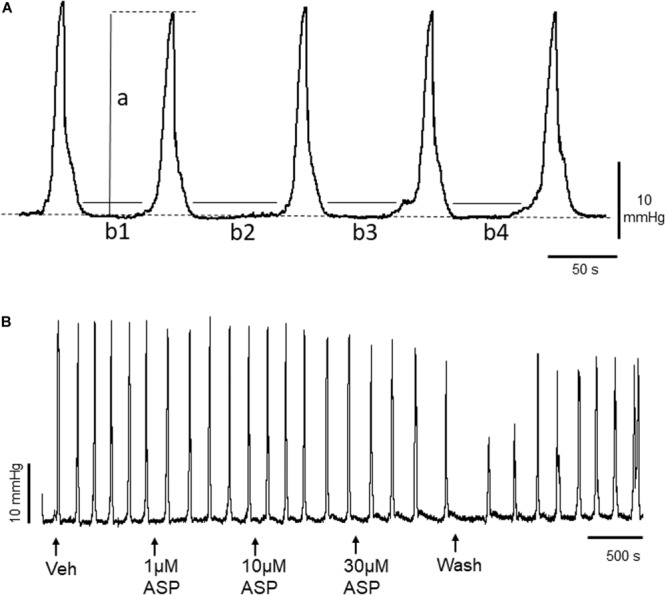
**(A)** Representative trace of basal murine colonic peristaltic motor complexes (CPMCs) recorded under basal conditions. The parameters used to evaluate changes in motility are illustrated; *a*, the amplitude of individual CPMCs, (*b^1^+b^2^+etc*), the time in quiescence (TIQ) which is the total time the tissue is at baseline activity during a 15 min (900 s) test period. The frequency represents the number of CPMC complexes measured during a 15 min time period. The dashed line represents baseline intraluminal pressure. **(B)** Effect of the TRPA1 agonist ASP7663 (ASP) upon CPMC activity. The trace shows a representative recording of the effects of cumulative additions of ASP7663 upon CPMC activity. ASP7663 reversibly decreased CPMC activity in a concentration-dependent fashion.

The methodology has been described in detail previously (Keating et al., 2010). Briefly, animals were euthanized as described above, and a midline laparotomy was immediately performed and the exposed abdomen bathed in Krebs solution [in mM: 119 NaCl, 4.7 KCl, 1 NaH_2_PO_4_, 1.2 MgSO_4_, 25 NaHCO_3_, 2.5 CaCl_2_, 11 glucose] gassed with carbogen [95% O_2_, 5% CO_2_]. The entire colon was removed and immediately placed into a beaker of carbogen-gassed Krebs solution. Using a syringe, the lumen was then cleared of any contents by gentle flushing with Krebs solution. An adapted standard organ bath procedure was used. Segments of colon (6 cm length) were placed into the organ bath (20 mL volume) perfused continuously (10 mL min^–1^) with 37°C Krebs solution, gassed with carbogen, and equilibrated to pH 7.4. The oral and aboral ends of the colon segment were securely attached to an input and output port of the organ bath, respectively. The input port was connected to a reservoir and syringe set-up which allowed the controlled perfusion of Krebs solution through the lumen of the colonic segment, whilst the output port was attached in series to a pressure transducer (BD DTXPlus^TM^, Oxford, United Kingdom). Motor activity was initiated in the colon segments by an infusion of Krebs from the syringe into the lumen of the colon until an intraluminal pressure of ~5 mmHg had been reached. Under these conditions, regular aborally propagating waves of contraction developed spontaneously and persisted over time. These contractile waves were recorded as changes in intraluminal pressure (Figure 1A) and were termed CPMCs, similar to the normal peristaltic activity of the colon. After an equilibration period of 40 min CPMC activity had reached a consistent pattern, in terms of their amplitude and frequency, and experimental procedures were then started. In a small number of experiments (~2%) tissues failed to show robust CPMC-like activity and these were discarded from use before any experimental procedures took place.

### *In vitro* Bioassay: Experimental Procedure

Compounds were bath applied using a cumulative concentration response protocol consisting of successive 15 min perfusion periods, starting with the vehicle and followed by 3 incremental concentrations of a drug, followed by a washout period to assess the reversibility of effects. In the experiments in which the TRPA1 antagonist HC-030031 was used to investigate the specificity of TRPA1 agonist responses, the antagonist was added to the bath for 15 min prior to the addition of the agonists.

### CPMC Activity Quantification

CPMC activity was quantified using a set of three parameters calculated for each 15 min experimental phase of the cumulative concentration experiments. These were: CPMC frequency (number of CPMCs/15 min), the time in quiescence (TIQ, s), which is a measurement of the total time that the tissue is at baseline activity during the 15 min period of drug or vehicle perfusion and the average CPMC amplitude (mmHg) (Figure 1A). The amplitude reflected the mechanical activity of the tissue, whereas the TIQ reflected the overall time with motor activity.

### Data Analysis

Changes in intraluminal pressure generated by CPMC activity were amplified (Digitimer, NL108, Welwyn Garden City, United Kingdom) and subsequently acquired to a computer through a CED 1401 interface and Spike2 software (Cambridge Electronic Design, Cambridge, United Kingdom). Intraluminal pressure was sampled at 100 Hz. Analysis was carried out off-line using the software applications contained in the Spike2 software package. Data for TIQ and amplitude were also expressed as percentage changes relative to their corresponding vehicles (relative changes) and were compared to relative changes in TIQ and amplitude calculated from time-matched vehicle control experiments.

### Statistical Analysis

Raw data are expressed as mean ± SEM; “n” refers to the number of animals from which the data was obtained. Statistical analysis was performed using repeat measures one-way analysis of variance (ANOVA) with Dunnet’s *post hoc* test as appropriate (Prism 7.0, Graphpad software, San Diego, CA, United States). Potential differences between agonist concentration response curves in the absence and presence of antagonists were determined using two-way ANOVA (Prism 7.0, Graphpad software, San Diego, CA, United States). Significance was achieved with a *p* < 0.05.

### Drugs

Compounds were obtained from Sigma (Dorset, United Kingdom), Tocris (Bristol, United Kingdom) and Enzo Biosciences (Switzerland). Stock solutions of 1 or 10 mM were prepared by adding an appropriate volume of either H_2_O or DMSO and were diluted to test concentrations in Krebs solution. 4-hydroxy-2-nonenal was prepared as instructed and stored in single aliquots at −80^o^C. Aliquots were thawed and diluted into Krebs solution immediately before use. The maximum concentration of DMSO used was 0.1%. Drugs were applied to the serosal surface and while the bath concentrations of each compound were not measured during the experiments, they were assumed to reach a steady-state concentration equal to that of the perfusate.

## Results

### Colonic Peristaltic Motor Complex (CPMC) Activity

CPMC activity was initiated in individual mouse colonic segments via luminal infusion of Krebs solution and was monitored experimentally as increases in intraluminal pressure (Figure 1A) which propagated in an aboral direction along the whole length of the colon. Baseline data on CPMC activity (collected over a 15 min period from a total of 51 experiments) are summarized as follows: frequency (the number of CPMCs/900 s), 8.2 ± 0.32; TIQ (time in quiescence), 563.4 ± 13 s; amplitude (CPMC amplitude), 33 ± 1.8 mmHg.

### The Effect of HC-030031 on CPMC Activity

To determine whether TRPA1 played a role in regulating baseline CPMC activity we tested whether CPMC activity could be affected by a TRPA1 antagonist. We tested the effect of the TRPA1 antagonist HC-030031(1–10 μM) on CPMC activity and found that HC-030031 had no significant effect upon baseline CPMC activity at any of the concentrations tested (Supplementary Figure S1).

### The Effect of ASP7663 on CPMC Activity

**FIGURE 2:**
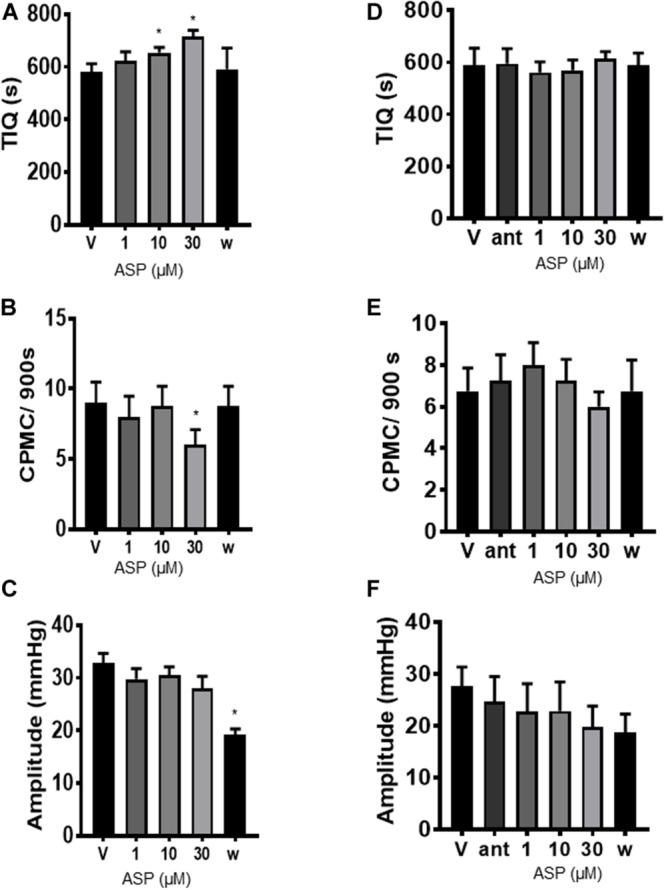
ASP7663 inhibited CPMC activity in mouse colonic segments. **(A–C)**. Graphs Illustrating the concentration dependent effects of ASP7663 (*n* = 4) on **(A)** TIQ **(B)** frequency, and **(C)** amplitude of CPMCs in mouse colonic segments. The effects on ASP7663 upon CMPC frequency and TIQ are reversible upon washout whereas ASP7663 irreversibly decreases CPMC amplitude. **(D–F)** Graphs illustrating the effects of HC-030031 (10 μM) on ASP7663 induced changes in **(D)** TIQ, **(E)** frequency, and **(F)** amplitude of CPMCs. HC-030031 (*n* = 4) blocks the effects of ASP7663 on CPMC activity. “ant” represents HC-030031, “V” represents vehicle, whilst “w” represents activity after 45 min washout period in this and all subsequent figures. Data are expressed as mean ± SEM; *p < 0.05 vs. **(A–C)** vehicle or **(D–F)** antagonist by repeated measures one-way ANOVA.

To determine whether TRPA1 agonists had any effect upon CPMC activity we tested a range of compounds using a cumulative dosing approach. ASP7663 (1–30μM) caused a concentration dependent decrease in CPMC activity (Figure 1B) shown as an increase in the TIQ (Figure 2A), a decrease in frequency (Figure 2B) and a decrease in CPMC amplitude (Figure 2C). It was noted that the effects of ASP7663 upon TIQ and frequency reversed upon washout whereas the amplitude changes were irreversible. Bath perfusion of HC-030031 (10 μM) blocked the effects of ASP7663 upon CPMC activity (Figures 2D–F).

### The Effect of Cinnamaldehyde on CPMC Activity

**FIGURE 3:**
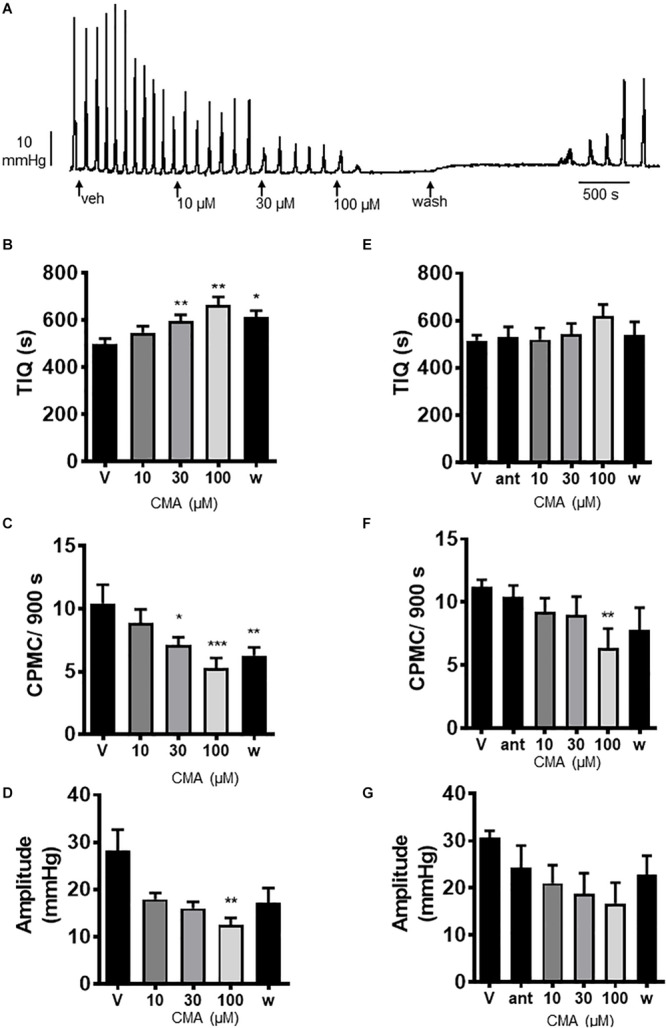
Cinnamaldehyde (CMA) inhibited CPMC activity. **(A)** Representative recording of the effects of cumulative additions of CMA upon CPMC activity. **(B–D)** Graphs illustrating the concentration dependent effects of CMA (*n* = 7) on **(B)** TIQ, **(C)** frequency, and **(D)** amplitude of CPMCs in isolated mouse colonic segments. CMA decreased CPMC activity in a concentration dependent fashion. The effects of CMA on frequency and TIQ do not reverse upon washout, whilst the inhibitory effects of CMA upon CPMC amplitude are reversible. **(E–G).** Graphs illustrating the effects of HC-030031 (10 μM) on CMA induced changes in **(E)** TIQ; **(F)** frequency, and **(G)** amplitude of CPMCs in isolated mouse colonic segments. HC-030031 (*n* = 5) attenuates the effects of CMA on CPMC activity. Data are expressed as mean ± SEM; *p < 0.05; **p < 0.01; ***p < 0.001 vs. **(B–D)** vehicle or **(E–G)** antagonist by repeated measures one-way ANOVA.

We next tested the TRPA1 activator cinnamaldehyde (CMA) for its effects on CPMC activity. CMA (10–100 μM) caused a concentration dependent decrease in CPMC activity (Figure 3A) in which both 30 and 100 μM CMA significantly increased TIQ (Figure 3B) and decreased frequency (Figure 3C), whilst CPMC amplitude was only decreased by 100 μM CMA (Figure 3D). The inhibitory effects of CMA upon CPMC frequency and TIQ were sustained into the late washout phase whereas the inhibitory effects of CMA upon CPMC amplitude reversed upon washout (Figure 3D). HC-030031 blocked the effects of CMA (Figures 3E–G) although in the presence of 10 μM HC-030031, 100 μM CMA still caused a significant reversible decrease in CPMC frequency (Figure 3F).

### The Effects of L-NAME on CMA Induced Changes in CPMC Frequency

**FIGURE 6:**
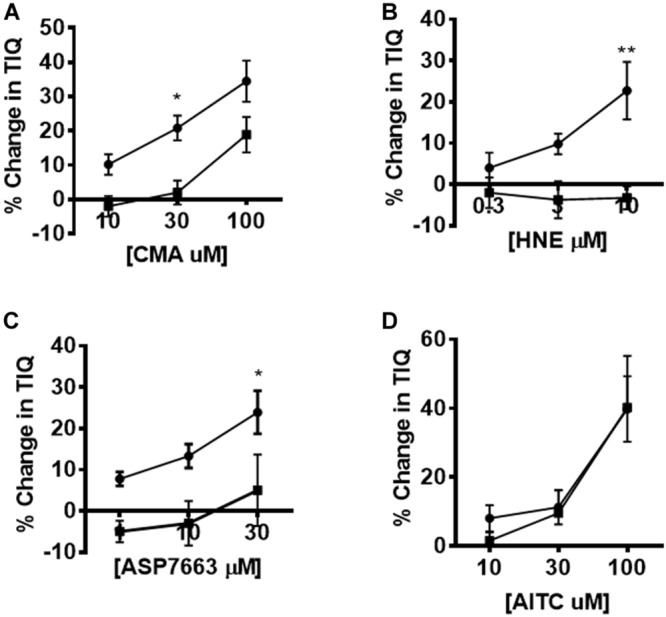
Pharmacological inhibition of agonist-induced changes in CPMC activity. The effect of HC-030031 on changes in TIQ induced by CMA, HNE, ASP7663 and AITC. Exposure to HC-030031 (10 μM) significantly decreased the dose response curves to **(A)** CMA, **(B)** HNE, **(C)** ASP7663. **(D)** Exposure to HC-030031 (10 μM) had no effect on the dose response curve to AITC. Data are expressed as mean ± SEM. Closed circles represent agonist alone and closed squares represent agonist + antagonist. Data are expressed as mean ± SEM; *p < 0.05. **p < 0.01. Curves were compared by using two-way ANOVA with Bonferroni *post hoc* tests.

Studies have demonstrated that TRPA1 channels are expressed on and activate inhibitory nitrergic neurons within the ENS (Poole et al., 2011) and as such we examined the effects of blocking nitrergic neurons upon CMA-evoked changes in CPMC. Firstly, we tested the effects of N^G^*-nitro-L-arginine methyl ester* (L-NAME) upon CPMC activity. L-NAME (100 μM) caused a concentration dependent and significant increase in CPMC activity, shown as a decrease in TIQ (Figure 6A) and an increased frequency (Figure 6B). L-NAME had no effect upon CPMC amplitude (Figure 6C). In the presence of 100 μM L-NAME, 10 μM, and 100 μM CMA had no significant effect upon CPMC activity (Supplementary Figure S2).

### The Effects of Allyl Isothiocyanate on CPMC Activity

**FIGURE 4:**
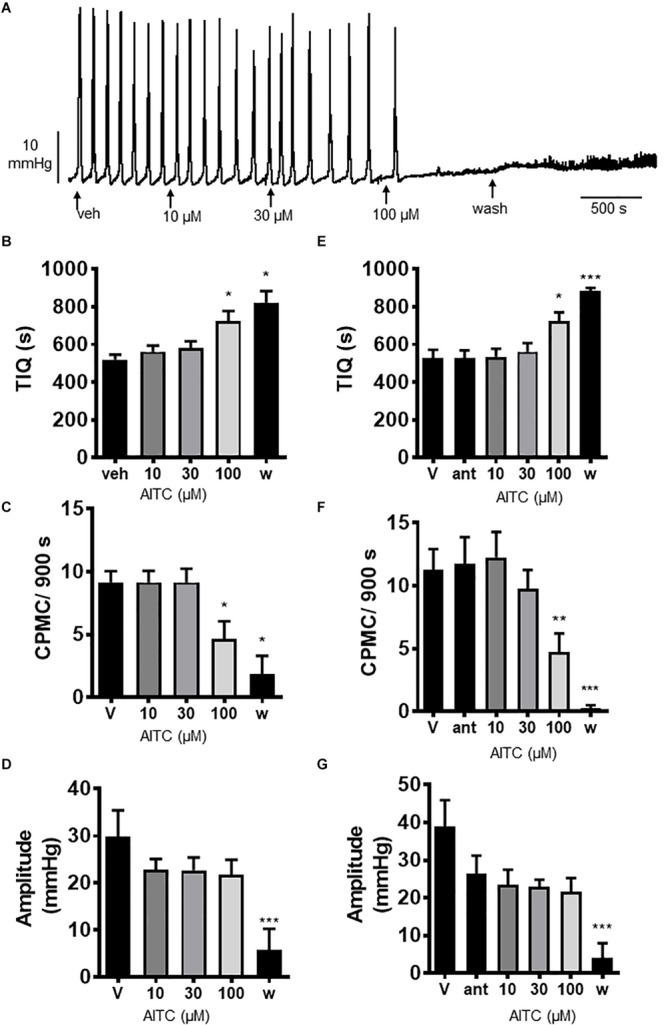
Allyl isothiocyanate blocked CPMC activity in the isolated colon of mice. **(A)** Representative recording of the effects of cumulative additions of allyl isothiocyanate (AITC) upon CPMC activity. **(B–D)** Graphs illustrating the concentration dependent effects of AITC (*n* = 6) on **(B)** TIQ **(C)** frequency and **(D)** amplitude of CPMCs. 10 μM and 30 μM AITC had no effect on CPMC activity, but 100 μM AITC decreased frequency and increased TIQ whilst having no effect upon CPMC amplitude. The effects of AITC on CPMC activity did not reverse upon washout and were not blocked by HC-030031 (10 μM). In the presence of 10 μM HC-030031, 100 μM AITC decreased CPMC frequency and increased the TIQ in an irreversible fashion. **(E–G)** Graphs illustrating the effects HC-030031 on AITC (n = 4) induced changes in **(E)** TIQ, **(F)** frequency, and **(G)** amplitude of CPMCs. Data are expressed as mean ± SEM; *p < 0.05; **p < 0.01; ***p < 0.001 vs. **(B–D)** vehicle or **(E–G)** antagonist by repeated measures one-way ANOVA.

We tested the effects of the TRPA1 activator allyl isothiocyanate (AITC) upon CPMC activity in isolated colonic segments. AITC (10–100 μM) had a complex pattern of effects on CPMC activity (Figures 4A–D). Ten and thirty micromolar AITC had no effect upon CPMC activity whereas 100 μM AITC caused an irreversible suppression of CPMC activity (Figures 4B–D). The effects of AITC did not reverse upon washout, and the tissues were unable to produce CPMC activity even after a 45 min washout. The effects of AITC on CPMC activity were not blocked by 10 μM HC-030031(Figures 4E–G).

### The Effect of 4-Hydroxy-2-Nonenal on CPMC Activity

**FIGURE 5:**
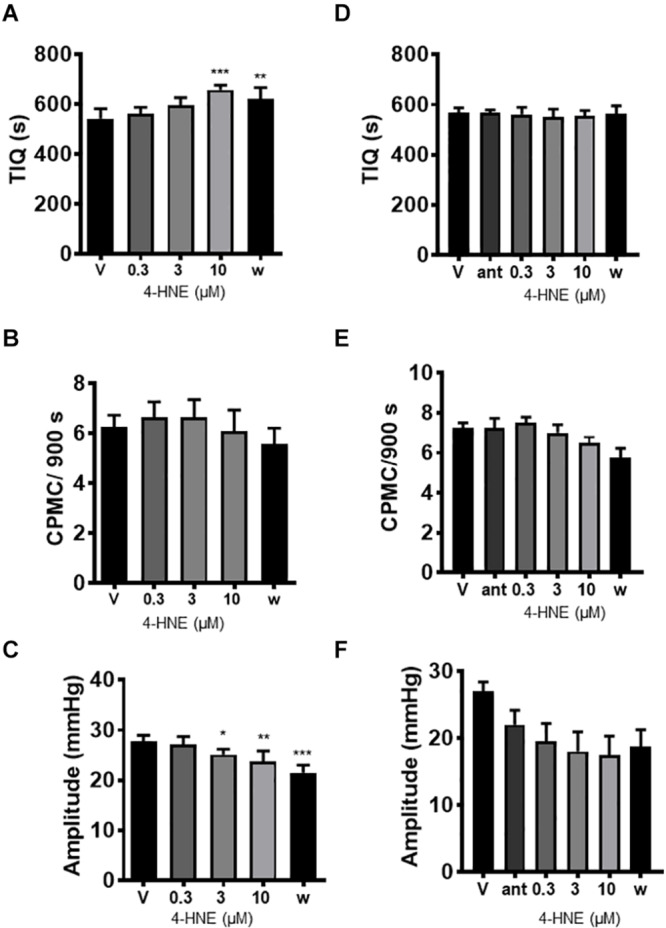
4-hydroxy-2-nonenal inhibited CPMC activity in isolated segments of mouse colon. In the presence of 4-hydroxy-2-nonenal (HNE) CPMC activity is decreased **(A–C)**. Graphs illustrating the effects of HNE (n = 4) on **(A)** TIQ, **(B)** frequency, and **(C)** amplitude of CPMCs. In the presence of HNE CPMC activity is decreased. **(D–F).** Graphs illustrating the effects HC-030031 on HNE (*n* = 4) induced changes in TIQ **(D)**; frequency **(E)**; and amplitude **(F)** of CPMCs. HC-030031 (10 μM) attenuates the effects of HNE on CPMC activity. Data are expressed as mean ± SEM; *p < 0.05; **p < 0.01; ***p < 0.001 vs. **(A–C)** vehicle or **(D–F)** antagonist by repeated measures one-way ANOVA.

The endogenous TRPA1 activator 4-hydroxy-2-nonenal (HNE) was assessed for its effects on CPMC activity. HNE (1–10 μM) caused a sustained concentration dependent decrease in CPMC activity shown as an increased TIQ (Figure 5A) although frequency was not significantly affected by HNE at any concentration tested (Figure 5B). Three and ten micromolar HNE decreased CPMC amplitude (Figure 5C). Bath perfusion of HC-030031 (10 μM) blocked the effects of HNE upon CPMC activity (Figures 5D–F). Changes in TIQ in the absence of changes in frequency could reflect altered CPMC width and as such we analyzed the CPMC width in the absence and presence of 10 μM HNE and found that the total CPMC width changed from 330 ± 25 s (vehicle) to 249 ± 17.6 s (HNE) (*p* = 0.01, *t*-test).

### Effects of HC-030031 on the Inhibitory Actions of TPRA1 Agonists Upon CPMC Activity

The effects of HC-030031 on TRPA1 agonist induced changes in TIQ was assessed by plotting the percentage changes in TIQ in the absence and presence of HC-030031. We found significant differences in the dose response curves for CMA, HNE and ASP7663 performed in the presence of 10 μM HC-030031 compared to the presence of vehicle (Figures 6A–C) (two-way ANOVA; *F* = 13.09, *p* = 0.001; *F* = 18.7, *p* = 0.0004; *F* = 15.3, *p* = 0.001 for CMA, HNE and ASP7663, respectively). However, the AITC induced changes in TIQ were not blocked by HC-030031 at any concentration of AITC tested (Figure 6D) (Two-way ANOVA; *F* = 0.16, *p* = 0.69).

### Effect of TRPA1 Agonists on Relative Changes in CPMC Activity

**FIGURE 7:**
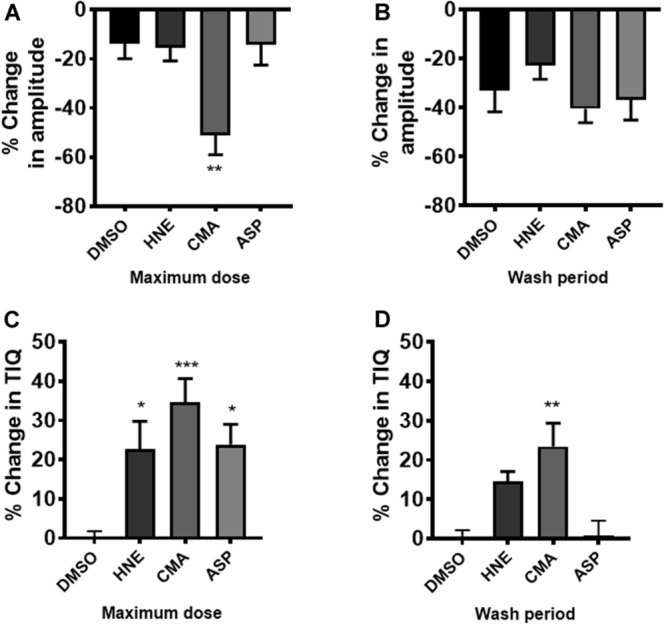
The effects of TRPA1 compounds on relative changes in TIQ and amplitude. CPMC amplitude for HNE, CMA, and ASP7665 were plotted as relative changes in amplitude at **(A)** their maximum dose compared to vehicle and **(B)** washout compared to vehicle. Compounds are plotted alongside appropriate time-matched vehicle control data (DMSO, *n* = 6). Similar analysis for TIQ in which relative TIQ values for the test compounds are plotted alongside appropriate time matched control data at **(C)** their maximum dose compared to vehicle and **(D)** washout compared to vehicle. Data are expressed as mean ± SEM; *p < 0.05; **p < 0.01; ***p < 0.001 vs. vehicle by one-way ANOVA.

The actions of ASP7663, CMA and HNE upon CPMC amplitude and TIQ were also plotted as percentage changes relative to their vehicle responses (relative change) and were then compared to appropriate time-matched controls (*n* = 6). At the highest drug dose tested only CMA caused a significant relative decrease in amplitude when compared against time- matched controls and relative amplitude changes reversed by washout (Figures 7A,B). Similar analysis for TIQ showed that all three compounds gave rise to significant relative TIQ increases, but by the washout phase the ASP7663 -induced relative increase in TIQ had reversed, whilst both HNE and CMA-induced relative TIQ increases were still elevated although only the CMA-induced effects were significant (Figures 7C,D).

## Discussion

This study set out to examine the role of TRPA1 activation in regulating colonic motility. We found that basal colonic motility behavior is not dependent upon TRPA1 activity, but that exogenous and endogenous TRPA1 agonists suppress colonic motor behavior. This supports a role for neuronal TRPA1 in regulating motor responses of the colon in response to dietary or inflammatory generated TRPA1 agonists.

We employed an *in vitro* model of peristaltic-like behavior (Abdu et al., 2002; Keating et al., 2014) to examine the role of TRPA1 in regulating colonic motility. CPMC activity in these preparations was invoked by a small distension of the colonic segments which gave rise to reproducible changes in contractile activity of the colon segments that were neurogenic and myogenic in origin. We focused on analyzing changes in amplitude, frequency and TIQ to assess the effects of TRPA1 compounds on CPMC activity. Measuring amplitude gave an indication of the compounds’ effects upon CPMC contractility, while TIQ and frequency assessed the motor pattern of CPMC activity (Keating et al., 2010). Our previous studies have demonstrated TIQ to be a robust and stable marker of CPMC frequency (Keating et al., 2010, 2014) reflecting the neuronal influence of the ENS upon motility behavior in these preparations and so we focused our analysis around this metric. As support for this we found that the nitric oxide synthase inhibitor L-NAME increased the TIQ of CPMC activity in these preparations reflecting the pharmacological and physiological actions of this compound on the ENS (Waterman and Costa, 1994; Rodríguez-Membrilla et al., 1995).

We were interested in assessing the role of TRPA1 channels in regulating CPMC activity. TRPA1 has a unique gastrointestinal expression pattern for a TRP channel in that as well as expression in sensory neurons (Brierley et al., 2009) epithelial cells (Kaji et al., 2012), and colonic mesenchymal cells (Yang et al., 2019), it is also localized to inhibitory motor neurons, descending interneurons, cholinergic neurons, and intrinsic primary afferent neurons of the ENS (Poole et al., 2011) which are subtypes of enteric neurons known to detect mechanical stimuli. Therefore TRPA1 channels may act as mechanosensory channels within enteric neurons controlling the activity of these neurons in response to mechanical stimulation such as distension of the gut. We found that in the presence of HC-030031 neither frequency nor amplitude of CPMCs were altered suggesting that TRPA1 plays no obvious mechanosensory role in normal CPMC activity. This supports data showing that TRPA1^–/–^ mice possess normal spontaneous motility (Poole et al., 2011) but contrasts with a mechanosensory role for TRPA1 proposed in spinal afferents whereby mechanically-induced inward currents in either DRG neurites or cell bodies were reduced by TRPA1 deletion or antagonism (Brierley et al., 2011). Furthermore TRPA1 channels have been shown to be active in mechanosensory signaling in extrinsic pathways in the gut (Brierley et al., 2009) and the bladder (Nicholas et al., 2017). It’s unlikely that the concentration of HC-030031 in these experiments was too low to have any significant effect upon basal CPMC activity. Whilst HC-030031 is a relatively weak antagonist of murine TRPA1 channels (pA_2_ ∼ 5.5; Ko et al., 2019) the antagonist was tested at an upper concentration shown to be specific for blocking TRPA1 (Capasso et al., 2012) and to cause almost complete inhibition of TRPA1 mediated Ca^2+^ currents in HEK cells expressing TRPA1 (McNamara et al., 2007).

If TRPA1 is not involved in regulating normal CPMC mechanosensory function, what are the activators of TRPA1 in the colon, and what are the effects of this activation upon CPMC activity? We investigated this by using a series of ligands with known actions on TRPA1. ASP7663, a synthetic TRPA1 agonist, attenuated CPMC activity, and these effects were inhibited by HC-030031. ASP7663 has been shown to increase Ca^2+^ currents in HEK cells expressing human, rat, and mouse TRPA1 channels, and stimulate 5-HT secretion from QGP-1 cells that are an EC cell lineage expressing TRPA1 channels (Kojima et al., 2014), thus demonstrating that this compound is a potent activator of TRPA1 channels. Our results revealed ASP7663 inhibited CMPC frequency, TIQ and amplitude that persisted into the washout phase. Furthermore, these changes were blocked by a TRPA1 antagonist suggesting that ASP7663 is acting via TRPA1 channels to mediate these inhibitory effects. However, we were mindful of over interpreting the data concerning drug-induced changes in amplitude as time-matched control experiments also revealed a small but significant time-dependent change in amplitude (Keating et al., 2014). This led to concerns that these time-dependent amplitude changes were confounding the drug-induced effects on this parameter, and so by plotting relative changes in CPMC amplitude and TIQ and comparing these to equivalent time-matched controls, we were able to control for these confounding factors. Analyzing the data in this fashion revealed that ASP7663 induced changes in CPMC activity only affected TIQ showing that activation of TRPA1 channels inhibits peristaltic-like behavior in the murine colon by acting on the neuronal mechanisms underlying motility behavior.

If CPMC activity can be attenuated using a synthetic TRPA1 agonist it would seem likely that exogenous alimentary agonists would act similarly. Therefore, we tested cinnamaldehyde (CMA), and allyl isothiocyanate (AITC) for their effects on CPMC activity. α, β- unsaturated aldehydes (CMA) and isothiocyanates (AITC) both activate TRPA1 channels through a process involving covalent modification of cysteine and lysine residues located within the amino-terminal cytoplasmic region of this channel. We found that CMA attenuated CPMC activity in an HC-030031 dependant fashion, and this was seen clearly in the rightward shift in the concentration response curve for CMA-induced changes in TIQ in the presence of HC-030031. This data identifies a role for TRPA1 induced signaling induced by an exogenous agonist to regulate distension induced motor activity in mouse colonic segments. We found that in contrast to the ASP7663 effects on TIQ that CMA-induced long-lasting changes in TIQ probably because modification of cysteine and lysine side chains by reactive aldehydes such as CMA are essentially irreversible (Aldini et al., 2007).

Our results with L-NAME demonstrated that the CMA mediated activation of TRPA1 inhibited colonic motor activity via a nitric oxide signaling pathway. TRPA1 channels have been shown to be expressed on inhibitory nitrergic neurons in the murine gastrointestinal tract (Poole et al., 2011) and their inhibition, and subsequent depression of nitric oxide synthesis, would have excitatory actions on CPMC activity through the removal of basal inhibitory tone. Inhibition of nitric oxide synthesis has been shown to have a prokinetic effect on gastrointestinal tract function (Waterman and Costa, 1994; Rodríguez-Membrilla et al., 1995), and here we found that L-NAME increased CPMC activity, but in the presence of L-NAME we found that the inhibitory effects of CMA upon CPMC activity were blocked suggesting that in this model CMA activates TRPA1 channels on nitrergic expressing motorneurons in the ENS to mediate inhibitory actions on motor activity.

AITC had complex effects upon CPMC activity, in which the lowest concentrations had no effect upon CPMC activity, whilst the highest concentration of AITC completely blocked CPMC activity. Furthermore, these inhibitory effects of AITC could not be reversed by HC-030031 which suggested that they were not being mediated through TRPA1. Although studies have shown that AITC activates gastrointestinal TRPA1 channels to mediate secretion and contraction of tissue (Fothergill et al., 2016; Yang et al., 2019) and that AITC can activate bladder afferents at similar concentrations to those used in this study (Nicholas et al., 2017) our results for AITC with HC-030031 supported no role for AITC in mediating CPMC activity via activation of TRPA1 channels. These results support previous data in which the inhibitory effects of AITC on intestinal contractility were also resistant to blockade by HC-030031 and were postulated to be due to AITC directly acting on smooth muscle (Capasso et al., 2012). Furthermore results from TRPA1^–/–^ animals showed that AITC activates calcium currents in myenteric neurons and mediates secretory responses (Poole et al., 2011; Fothergill et al., 2016) further demonstrating that AITC has small but viable off target effects within the gastrointestinal tract. It is worth noting that AITC at concentrations of 100 μM has been shown to be an agonist at TRPV1 receptors and therefore exposure to the maximum dose of AITC in these experiments could have desensitized TRPV1 channels expressed in the ENS, which could account for the loss of CPMC activity that we observed. Further we are mindful that intact colonic segments contain functional extrinsic afferent terminals that express TRPV1 and TRPA1. Activation of these mechanosensitive extrinsic afferents not only invokes central signaling processes, but also localized responses that have been implicated in controlling motor reflexes. It’s tempting to speculate that perhaps desensitization of these mechanosensitive extrinsic afferents accounted for the loss of CPMC activity in preparations exposed to elevated AITC concentrations. Like TRPV1 channels TRPA1 desensitize when exposed to agonists such as CMA or AITC (Akopian et al., 2007), and as such the results obtained with CMA and AITC could be due to direct desensitization of TRPA1. However we found that concentrations of AITC that have been shown to activate TRPA1 had no effect upon CPMC activity, whilst low concentrations of CMA decreased CPMC activity in an HC-030031 dependent fashion suggesting that modification of CPMC activity by lower concentrations of CMA was through direct actions on TRPA1 channels expressed by inhibitory neurons within the ENS.

We next asked whether endogenous activators of TRPA1 would affect CPMC activity and to explore this question we used 4-hydroxy-2-nonenal (HNE). Like CMA this compound is an α, β-unsaturated aldehyde but HNE is an endogenous molecule formed as a result of peroxidation of membrane phospholipids in response to tissue injury, inflammation, and oxidative stress (Esterbauer et al., 1991). Injections of HNE into rodent hind paws have been shown to elicit long-lasting pain-related behaviors that were inhibited by TRPA1 antagonists, and are absent in TRPA1^–/–^ animals demonstrating that HNE acts through a TRPA1 channel mediated response (Trevisani et al., 2007). We found that in addition to its role in promoting pain-like behavior, HNE inhibited CPMC activity in a similar long-lasting fashion specifically affecting TIQ when analyzed in terms of relative changes in CPMC activity. This suggested that motor activity in the colon could be regulated through the synthesis of endogenous inflammatory markers acting via TRPA1 and prompts consideration for an important role for TRPA1 within the ENS as a sensor for inflammation.

The unique expression profile of TRPA1 in the intestine suggests that signaling through this channel regulates multiple processes. TRPA1 expression in enteric neurons clearly regulates motility behavior via activation of inhibitory neurons whilst mucosal expression of TRPA1 on enteroendocrine cells in the small intestine signals the presence of nutrients within the gut (Cho et al., 2014). TRPA1 expression is also found in colonic enterocytes and mesenchymal cells where its activation can induce ion secretion and smooth muscle contraction via prostaglandin mediated processes (Kaji et al., 2012; Yang et al., 2019). A role for TRPA1-mediated responses to inflammation was proposed in a recent study showing that dextran sodium sulfate (DSS) invoked inflammation of the rat colon led to both an increased expression of HNE and abnormal colonic contractions which could be attenuated with a TRPA1 antagonist (Yang et al., 2019).

These results beg the question as to why we didn’t see any elevated contractile events in our experiments. A simple explanation for this is that our model measures a change in intraluminal pressure caused by neurogenically orchestrated waves of muscle contraction moving along the colonic segment and as such our model would record changes in smooth muscle contractility *per se* or increased secretion into the lumen as increases in baseline intraluminal pressure which interestingly we did see occur in preparations upon exposure to TRPA1 agonists such as HNE although the changes were not significantly different from control values. Also our drugs are added to the bathing solution surrounding the tissue and diffuse into the tissue via the serosa and consequently will reach the myenteric plexus before the mucosa.

The role of mucosal TRPA1 signaling may be to detect and respond to inflammation within the colon, but studies in *Drosophila* have shown that colonization of the intestine by pathogenic bacteria can activate contractions of the *Drosophila* gut via a TRPA1 mechanism which may serve a protective response to rid the intestine of harmful bacteria (Benguettat et al., 2018). It would be interesting to see whether this novel pathway is replicated in the mammalian gut and so would also implicate TRPA1 as a sensor for the gastrointestinal microbiome.

In conclusion we have used an *in-vitro* model of colonic motor activity to investigate the role of TRPA1 in regulating colonic motility and found that TRPA1 channels play no role in regulating basal distension induced motor activity in the mouse colon, but when activated by either endogenous or exogenous agonists acts in an inhibitory fashion to preferentially decrease the frequency of CPMC activity. The endogenous ligands that drive this process and the physiological signaling pathways that underpin this channel’s function are a target for further research, but it may well be that one of the roles of enteric TRPA1 activation is as a danger signal to reduce motility, and that inflammatory and or microbiome signals could be the stimuli to initiate this pathway.

## Data Availability Statement

The datasets generated for this study are available on request to the corresponding author.

## Ethics Statement

The animal study was reviewed and approved by the University of Hertfordshire, animals were maintained in accordance with Home Office regulations following the Animal and Scientific Procedures Act 1986.

## Author Contributions

A-AH, BS, and ZC performed most of the experiments and analyzed the data. A-AH contributed to writing the manuscript. CK designed the project, performed some of the experiments, and wrote the manuscript.

## Conflict of Interest

The authors declare that the research was conducted in the absence of any commercial or financial relationships that could be construed as a potential conflict of interest.

## Supplementary Material

FIGURE S1 Effects of HC-030031 upon baseline CPMC activity in the isolated colon of mice. **(A)** Representative recording showing the effects of **(Ai)**, vehicle and **(Aii)** 10μM HC-030031 upon CPMC activity. CPMC activity is unchanged in response to application of HC-030031. **(B–D)** Graphs illustrating the concentration dependent effects of HC-030031 (*n* = 5) on **(B)** TIQ, **(C)** frequency, and **(D)** amplitude of CPMCs. Data are expressed as mean ± SEM. In all experiments there was no significant difference between the CPMC parameters recorded in the presence of vehicle or HC-030031 (repeat measures one-way ANOVA).

FIGURE S2 Effects of L-NAME on CMA induced changes in CPMC activity in isolated segments of mouse colon. In the presence of L-NAME CPMC activity is unchanged in response to application of CMA. **(A–C)**. Graphs illustrating the concentration dependent effects of CMA in the presence of L-NAME (*n* = 5) on the **(A)** TIQ, **(B)** frequency, and **(C)** amplitude of CPMCs. Data are expressed as mean ± SEM; **p* < 0.05; ***p* < 0.01 vs. L-NAME by repeated measures one-way ANOVA.

## Abbreviations

AITC: allyl isothiocyanate
CMA: cinnamaldehyde
CPMCs: colonic peristaltic motor complexes
DSS: dextran sodium sulfate
EEC: enteroendocrine cell
EC: enterochromaffin
GI: gastrointestinal
HEK: human embryonic kidney
HNE: 4-hydroxy-2-nonenal
IP: intraluminal pressure
L-NAME: *N*^G^-nitro-L-arginine methyl ester
TIQ: time in quiescence
TRP: transient receptor potential
TRPA1: transient receptor potential ankyrin 1
TRPA1-IR: TRPA1 immunoreactivity.
