# Supplementary figures and images for: TRPA1 Channel Activation Inhibits Motor Activity in the Mouse Colon
Source: Front Neurosci. 2020 May 27;14:471. doi: 10.3389/fnins.2020.00471 (PMC7267031; doi:10.3389/fnins.2020.00471)

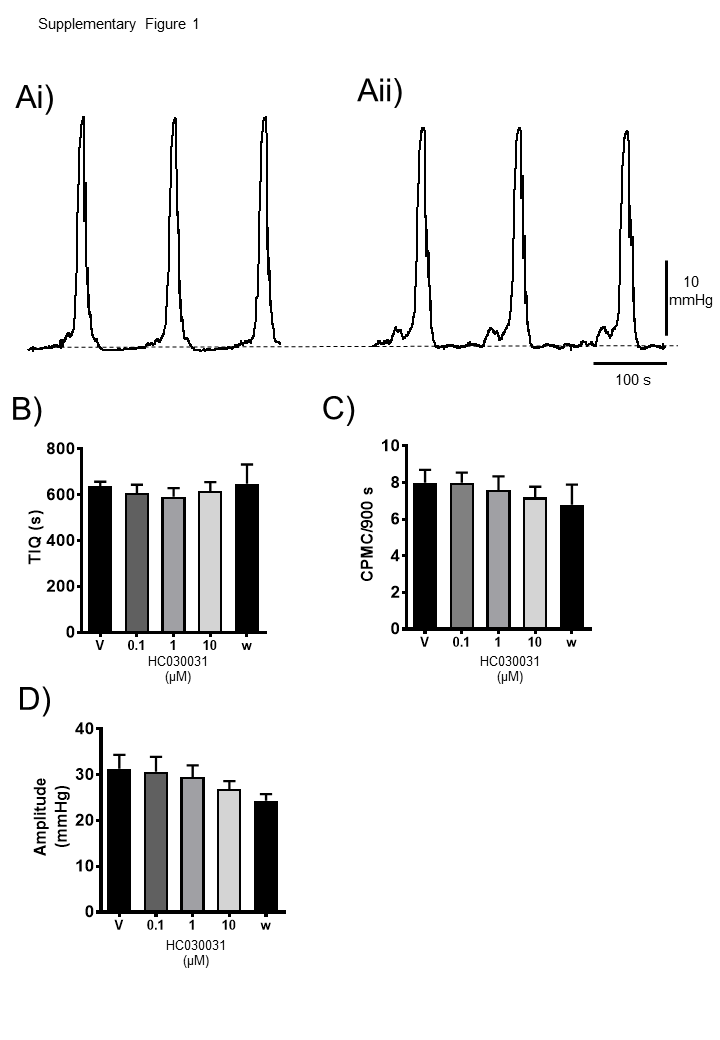

Supplement: FIGURE S1 — Effects of HC-030031 upon baseline CPMC activity in the isolated colon of mice. (A) Representative recording showing the effects of (Ai), vehicle and (Aii) 10μM HC-030031 upon CPMC activity. CPMC activity is unchanged in response to application of HC-030031. (B–D) Graphs illustrating the concentration dependent effects of HC-030031 (n = 5) on (B) TIQ, (C) frequency, and (D) amplitude of CPMCs. Data are expressed as mean ± SEM. In all experiments there was no significant difference between the CPMC parameters recorded in the presence of vehicle or HC-030031 (repeat measures one-way ANOVA). [file Image_1.tif]

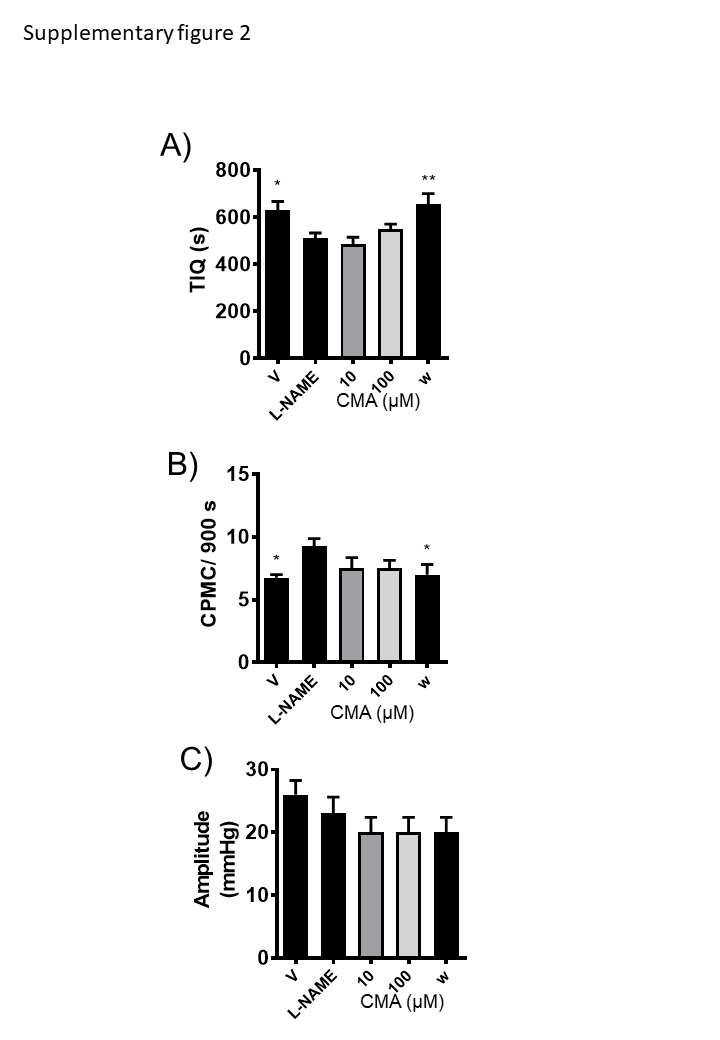

Supplement: FIGURE S2 — Effects of L-NAME on CMA induced changes in CPMC activity in isolated segments of mouse colon. In the presence of L-NAME CPMC activity is unchanged in response to application of CMA. (A–C). Graphs illustrating the concentration dependent effects of CMA in the presence of L-NAME (n = 5) on the (A) TIQ, (B) frequency, and (C) amplitude of CPMCs. Data are expressed as mean ± SEM; *p < 0.05; **p < 0.01 vs. L-NAME by repeated measures one-way ANOVA. [file Image_2.tif]
